# Intraoperative Diaphragmatic Plication During Initial Surgery With Phrenic Nerve Resection

**DOI:** 10.1093/icvts/ivaf233

**Published:** 2025-09-25

**Authors:** Tomomi Isono, Mitsunori Ohta, Ryu Kanzaki, Jiro Okami, Yasunobu Funakoshi, Seiji Taniguchi, Yoshihisa Kadota, Kensuke Kojima, Toshiteru Tokunaga, Satoshi Kawanaka, Yukiyasu Takeuchi, Hidenori Kusumoto, Hiroyuki Shiono, Hideoki Yokouchi, Teruo Iwasaki, Naoki Ikeda, Naoko Ose, Yasushi Shintani

**Affiliations:** Department of General Thoracic Surgery, Graduate School of Medicine, the University of Osaka, Osaka 565-0871, Japan; Department of General Thoracic Surgery, Shion Hospital, Osaka 557-0034, Japan; Department of General Thoracic Surgery, Osaka International Cancer Institute, Osaka 540-0008, Japan; Department of General Thoracic Surgery, Osaka International Cancer Institute, Osaka 540-0008, Japan; Department of General Thoracic Surgery, Osaka General Medical Center, Osaka 558-8558, Japan; Department of General Thoracic Surgery, Osaka Habikino Medical Center, Osaka 583-0872, Japan; Department of General Thoracic Surgery, Osaka Habikino Medical Center, Osaka 583-0872, Japan; Department of General Thoracic Surgery, National Hospital Organization Kinki-Chuo Chest Medical Center, Osaka 591-8555, Japan; Department of General Thoracic Surgery, National Hospital Organization Kinki-Chuo Chest Medical Center, Osaka 591-8555, Japan; Department of General Thoracic Surgery, NHO Osaka Toneyama Medical Center, Osaka 560-8552, Japan; Department of General Thoracic Surgery, NHO Osaka Toneyama Medical Center, Osaka 560-8552, Japan; Department of General Thoracic Surgery, Toyonaka Municipal Hospital, Osaka 560-8565, Japan; General Thoracic Surgery, Kindai University Nara Hospital, Nara 630-0293, Japan; Department of Surgery, Suita Municipal Hospital, Osaka 564-8567, Japan; Department of General Thoracic Surgery, Japan Organization of Occupational Health and Safety, Osaka Rosai Hospital, Osaka 591-8025, Japan; Department of General Thoracic Surgery, Naha City Hospital, Okinawa 902-0061, Japan; Department of General Thoracic Surgery, Graduate School of Medicine, the University of Osaka, Osaka 565-0871, Japan; Department of General Thoracic Surgery, Graduate School of Medicine, the University of Osaka, Osaka 565-0871, Japan

**Keywords:** phrenic nerve, diaphragm plication, lung cancer, thymic tumour

## Abstract

**Objectives:**

Diaphragmatic palsy can result in respiratory failure, potentially alleviated by diaphragmatic plication. Nevertheless, the benefits of preventive plication during phrenic nerve resection remain uncertain. This study evaluated whether preventive plication during primary surgery involving phrenic nerve resection alleviate paradoxical diaphragmatic movement and pulmonary function loss.

**Methods:**

Among 24,527 surgeries for lung cancer or mediastinal tumours at 11 institutions, 142 involved phrenic nerve resections. Of these, 132 patients were retrospectively analysed. Diaphragmatic displacement and pulmonary function were assessed pre- and postoperatively. Displacement was quantified by measuring thoracic height on pre- and postoperative chest X-rays (*D*, *D*′). Diaphragmatic displacement ratio was defined as: DDR = (*D*′ − *D*)/*D* × 100.

**Results:**

Seventy patients (53%) underwent preventive diaphragmatic plication during the primary surgery; 62 (47%) did not. Differences were significant overall and more pronounced in those undergoing left lobectomy or more extensive resection. In this subgroup, plication was associated with a smaller change in DDR (−30.1 ± 7.7% vs −20.2 ± 7.7%, *P* = .002), and smaller declines in % predicted forced vital capacity (−30.5 ± 8.0% vs −16.8 ± 17.7%, *P* = .029) and forced expiratory volume in 1 second (−31.6 ± 11.0% vs −19.0 ± 14.5%, *P* = .046).

**Conclusions:**

In patients undergoing left lobectomy or more extensive resections involving phrenic nerve resection, intraoperative diaphragmatic plication may help preserve postoperative pulmonary function. However, due to the small sample size and limited generalizability, these findings should be interpreted cautiously.

## Graphical abstract

**Figure:**
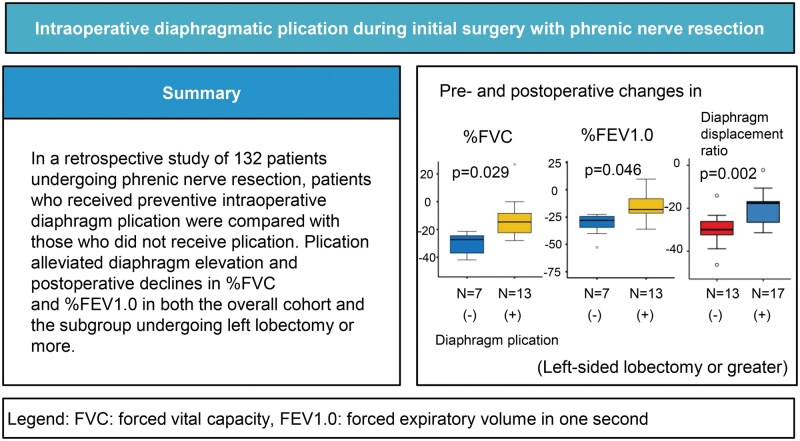

## INTRODUCTION

Phrenic nerve resection is sometimes necessary for complete tumour resection; however, it often results in postoperative dyspnoea due to diaphragmatic palsy. Paradoxical diaphragmatic movement can lead to mediastinal shift and atelectasis, resulting in ventilation-perfusion mismatch and reduced pulmonary functions.^1–4^ When conservative management fails, diaphragmatic plication can improve pulmonary function by correcting atelectasis and improving parameters such as vital capacity by 9%, forced expiratory volume in 1 second (FEV1.0) by 8%-43%, forced vital capacity (FVC) by 3%-40%, and total lung capacity (16%-19%).^5–10^ In a study conducted by Celik et al.,^7^ most patients reported symptomatic relief and functional improvement after plication.

Despite these potential benefits, prophylactic plication during phrenic nerve resection is rarely performed, and supporting evidence is limited.^11–18^ Tokunaga et al.^12^ reported near-predicted postoperative vital capacity of 88.2% and FEV1.0 of 98.5% in 8 cases. Beattie et al.^14^ performed a systematic review in oncologic 37 cases (including 13 cases in Tokunaga’s study and 20 cases of pneumonectomy) and found that prophylactic or early diaphragmatic plication at the time of phrenic nerve transection resulted in postoperative FEV1.0 values at 86%-98% of predicted and FVC at 82%-89%, with a marked reduction in dyspnoea incidence. However, previous studies were limited by small sample sizes and the lack of direct control-group comparisons. We therefore conducted a multicentre retrospective study to evaluate the effects of diaphragmatic plication on pulmonary function, clinical outcomes, and prognosis.

## PATIENTS AND METHODS

### Ethics statement

The Institutional Review Boards approved the study protocol of the Ethics Committee of Osaka University Hospital (control number 22260, approval date: October 05, 2022) and those of the participating hospitals, which waived the requirement for informed consent based on the study’s retrospective observational design.

### Patients’ selection

**Figure 1.**
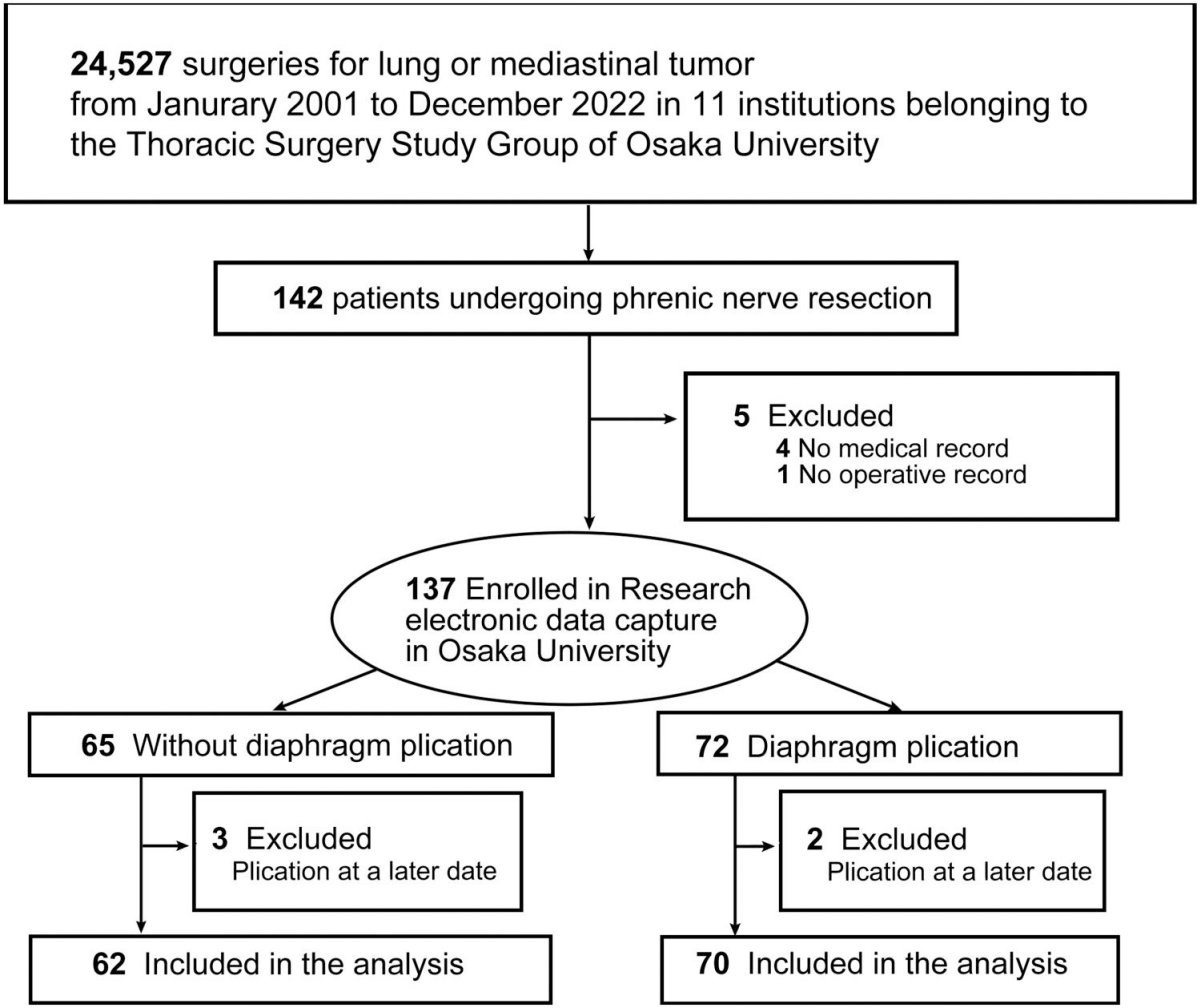
Patient Selection

From January 2001 to December 2022, 24,527 thoracic surgeries for lung cancer or mediastinal tumours were performed at 11 Osaka University Thoracic Surgery Study Group institutions. Phrenic nerve resection was conducted in 142 cases; after excluding 10 (5 with incomplete records, 5 with plication at a later date), 132 were included in the analysis (**Figure 1**).

### Diaphragmatic plication

Instrument selection, plication location, and tension varied by case. The basic technique involved imbricating the central portion of the diaphragm. In cases where multiple folds were plicated, the folds were sometimes secured by tying sutures between them (**Figure S1**). There was no specific protocol or set criteria for indicating plication. The decision was based on intraoperative judgement.

### Data collection

Clinical data were collected using Research Electronic Data Capture,^19^ hosted at Osaka University with secure login and audit trails. The variables included demographics, medical and surgical history, postoperative complications, radiologic findings, pulmonary function data, and survival outcomes. Diaphragmatic displacement was measured on chest radiographs by assessing the apex-to-diaphragm distance on the surgical side (*D* preoperatively and *D*′ postoperatively; **Figure S2**).

### Outcomes

The primary outcome was the change in pulmonary function, comparing measurements from 75 days before to the day of surgery (preoperative) with those ≥180 days later (postoperative).^20^ d%FVC (change in percent predicted forced vital capacity before and after surgery) and d%FEV1.0 (change in percent predicted forced expiratory volume in 1 second before and after surgery) were defined as differences (postoperative minus preoperative) in %FVC and %FEV1.0, respectively. Secondary outcomes included diaphragmatic displacement ratio (DDR) (DDR = (*D*′ − *D*)/*D* × 100) (%), complications, and 30-day survival.

### Statistical analysis

All statistical analyses were conducted using R version 4.2.2 (R Foundation for Statistical Computing, Vienna, Austria). Statistical significance was defined as *P* < .05. Continuous variables are reported as mean ± standard deviation, while categorical variables are presented as frequencies (%). Between-group comparisons used the *t*-test or Wilcoxon test for continuous variables based on normality and Fisher’s exact test for categorical variables. Preoperative and postoperative values were compared using paired *t*-tests. Post hoc power analysis was performed using observed effect sizes (Cohen’s *d*) for d%FVC and d%FEV1.0; multivariate linear regression adjusted for surgical approach, side, disease type, and resection extent.

### Guidelines

This study adhered to the recommendations outlined in the Strengthening the Reporting of Observational Studies in Epidemiology statement.^21^

## RESULTS

### Patients

Seventy patients (53%) underwent preventive diaphragmatic plication during the primary surgery, while 62 patients (47%) did not (**Figure 1**). Except for the surgical approach, patient profiles did not differ significantly between groups (**Table 1**). The without-plication group had more anterolateral/lateral/anterior axillary thoracotomies, while the plication group had more posterolateral and hemi-clamshell incisions.

**Table 1.** Patient Characteristics

	Without plication (*N* = 62)	Plication (*N* = 70)	*P*-value
Side			.147
Right	27 (44%)	21 (30%)	
Left	35 (56%)	49 (70%)	
Primary disease, no. (%)			.177
Lung cancer	17 (27%)	14 (20%)	
Thymic tumour	43 (69%)	56 (80%)	
Other	2 (3%)	0 (0%)	
Approach, no. (%)			<.001
VATS or RATS	4 (6%)	3 (4%)	
Anterolateral/lateral/anterior axially thoracotomy	17 (27%)	8 (11%)	
Posterolateral thoracotomy	1 (2%)	5 (7%)	
Sternotomy	29 (47%)	30 (43%)	
Hemi-Clamshell	6 (10%)	24 (34%)	
Trap door	4 (6%)	0 (0%)	
Other	1 (2%)	0 (0%)	
Resection of lung, no. (%)			.700
None	15 (24%)	10 (14%)	
Partial	19 (31%)	23 (33%)	
Segmentectomy	1 (2%)	1 (1 %)	
Lobectomy	23 (37%)	31 (44%)	
Pneumectomy	4 (6%)	5 (7%)	

The Fisher’s exact test was performed.

Abbreviations: RATS, robot-associated thoracic surgery; VATS, video-associated thoracic surgery.

### Postoperative course

Atrial fibrillation occurred more frequently in patients without plication. Only one patient in the plication group died within 30 days postoperatively (**Table 2**). No significant differences were observed between the 2 groups in other parameters, including postoperative survival rates during the 5-year follow-up period (*P* = .45, data not shown).

**Table 2.** Complications

	Without plication (*N* = 62)	Plication (*N* = 70)	*P*-value
Complications, no. (%)			
Pneumonia	2 (3%)	5 (7%)	.447
Atrial fibrillation	12 (20%)	4 (6%)	.030
Atelectasis	1 (2%)	0 (0%)	.470
Prolonged discharge (>POD30)	6 (10%)	10 (14%)	.594
Dead	0 (0%)	1 (1%)	1.0
Other	10 (16%)	18 (26%)	.205

Fisher’s exact test was performed.

Abbreviation: POD, postoperative date.

### Diaphragmatic displacement ratio

**Figure 2.**
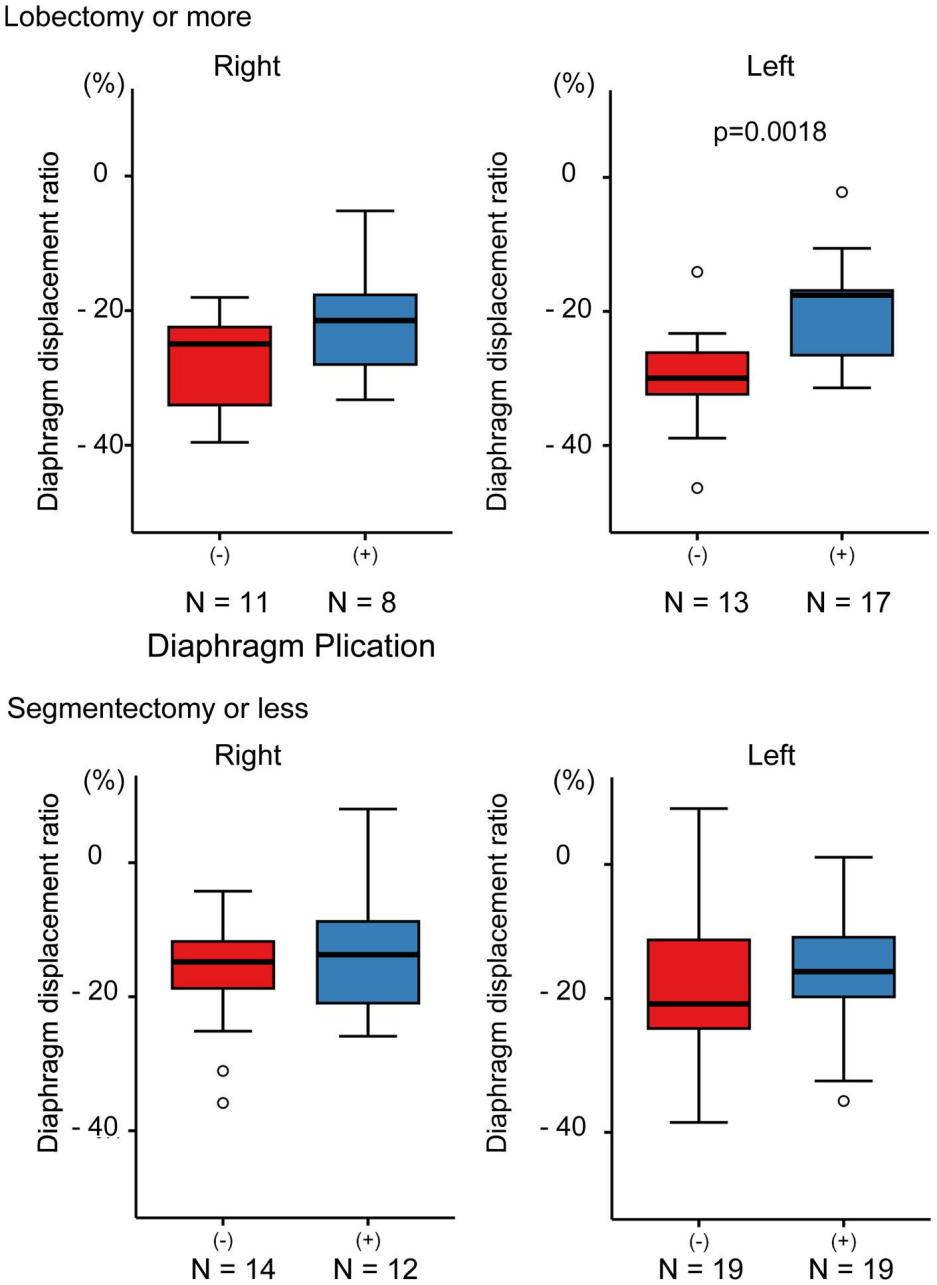
Subgroup Analysis of Diaphragm Displacement Ratio (DDR). DDR was calculated as: (postoperative thoracic height on chest X-rays − preoperative thoracic height)/preoperative thoracic height × 100 (%). The box boundaries represent the 25th and 75th percentiles, and the horizontal line indicates the median. Whiskers mark the minimum and maximum non-outlier values. White dots are outliers. (−) Indicates without-plication group; (+) indicates plication group. A *t*-test was used.

DDR showed significantly smaller change in the plication group than without plication (without-plication vs plication: −21.0 ± 12.8% vs −17.5 ± 9.4%, *P* = .035, **Figure S3**). In the subgroup that underwent left lobectomy or more extensive resections, the difference in DDR was also significant (−30.1 ± 7.7% vs −20.2 ± 7.7%, *P* = .002, **Figure 2**).

### Pulmonary function

**Figure 3.**
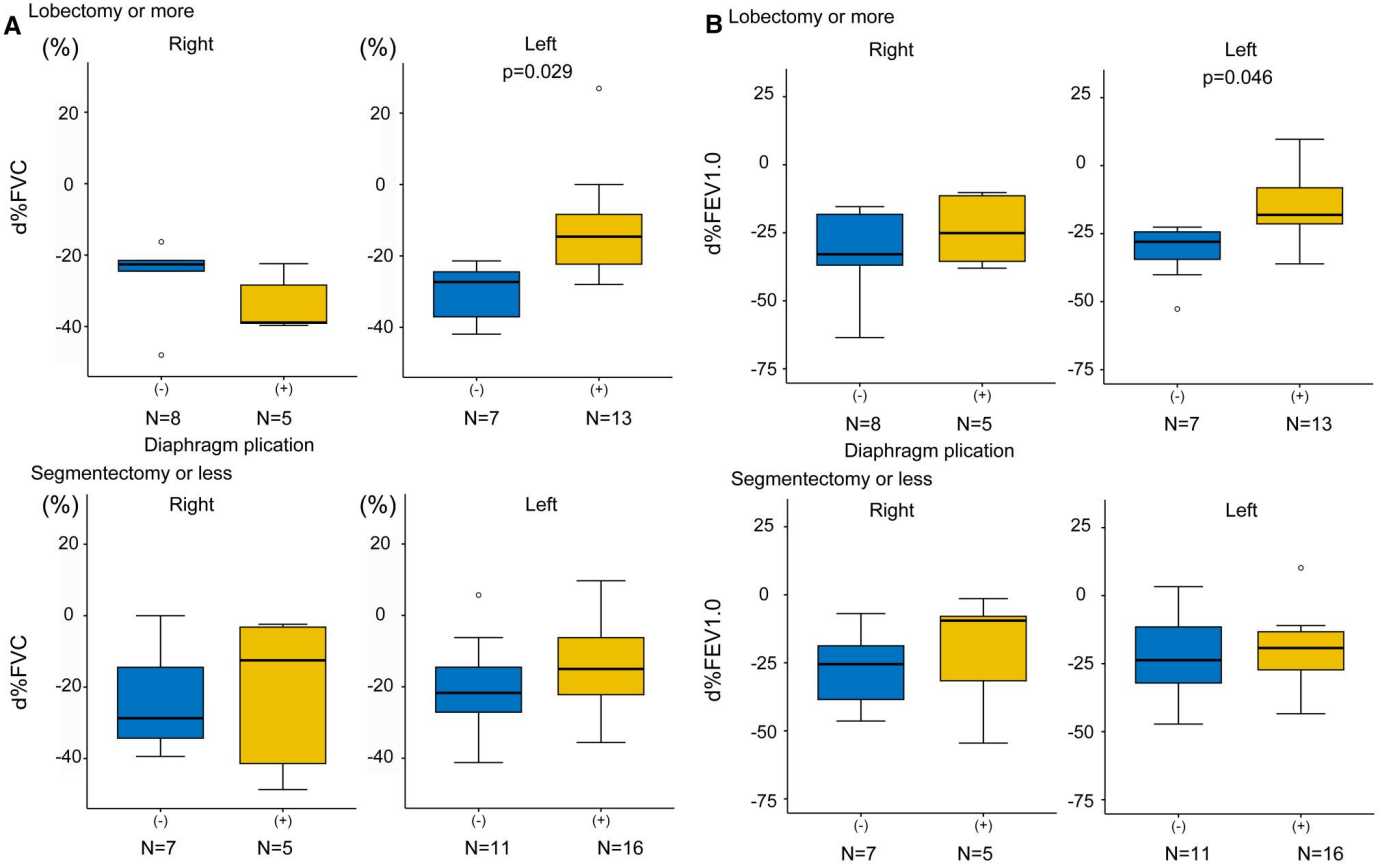
Subgroup Analysis of (A) d%FVC and (B) d%FEV1.0. d%FVC represents the change in percent predicted forced vital capacity (postoperative − preoperative), while d%FEV1.0 denotes the change in percent predicted forced expiratory volume in 1 second (postoperative − preoperative). The box boundaries represent the 25th and 75th percentiles, with the horizontal line indicating the median. Whiskers mark the minimum and maximum non-outlier values. White dots represent outliers. (−) indicates without-plication group; (+) indicates plication group. A *t*-test or Wilcoxon test was applied based on normality.

Among the 132 patients, preoperative pulmonary function test results were available for 126 patients (95%), and postoperative results for 74 patients (56%). Both preoperative and postoperative data were available for 72 patients (55%). There were 2 patients for whom only postoperative data were available, as their preoperative data could not be obtained. Postoperative pulmonary function tests were conducted at a mean of 25.6 ± 17.6 months without plication compared to 40.4 ± 31.3 months with plication (*P* = .02). Postoperative pulmonary function significantly declined compared to preoperative values across most subgroups (**Figure S4**). In the overall cohort, the plication group demonstrated significantly better d%FVC (−25.7 ± 13.3% vs −18.5 ± 16.1%, *P* = .041, **Figure S5**) and d%FEV1.0 (−28.4 ± 14.7% vs −20.7 ± 14.3%, *P* = .027, **Figure S6**). Subgroup analysis of patients undergoing left lobectomy or more extensive resections revealed even greater differences favouring plication, with improved d%FVC (−30.5 ± 8.0% vs −16.8 ± 17.7%, *P* = .029; **Figure 3A**) and d%FEV1.0 (−31.6 ± 11.0% vs −19.0 ± 14.5%, *P* = .046; **Figure 3B**). The qualitative results remained consistent when analyses were repeated excluding pneumonectomy cases (**Figures S7–S10**) and when limited to thymic epithelial tumours only (**Figures S11–S14**).

### Post hoc power analysis and multivariate analysis

In subgroup analysis of patients undergoing left lobectomy or more extensive resections, Power was 0.447 for d%FVC (d = 0.91) and 0.471 for d%FEV1.0 (*d* = 0.94). In the adjusted model, plication remained associated with a 6.47% smaller decline in d%FVC (*β* = –6.47, *P* = .087) and a 7.11% smaller decline in d%FEV1.0 (*β* = –7.11, *P* = .062) (**Table S1**).

## DISCUSSION

This study examined preventive diaphragmatic plication’s effects during phrenic nerve resection. The key finding is that preventive diaphragmatic plication might alleviate diaphragm displacement and the decline in FVC and FEV1.0 in left lobectomy or more extensive resections.

In left lobectomy or larger resections, the plication group had significantly higher %FVC and %FEV1.0, suggesting additional improvements in pulmonary function. DDR was significantly smaller in plication group than without, indicating effective diaphragmatic plication. In contrast, for segmentectomy or smaller resections, no significant differences were observed irrespective of side. However, a trend towards benefit was noted, particularly for left-sided segmentectomy or less resections (d%FVC: −20.3 ± 13.1% vs −14.2 ± 12.6%, *P* = .24). Based on this variance, a sample size of 73 patients per group would be required to achieve statistical significance, making it difficult to draw definitive conclusions. Nevertheless, plication may help mitigate postoperative declines in pulmonary function even after limited resections on the left side.

No significant benefit in pulmonary function was observed after right phrenic nerve resection, likely because the liver stabilizes the right hemidiaphragm and reduces paradoxical motion, thereby limiting the impact of plication on lung volumes. Even in the absence of significant spirometric gains, diaphragmatic plication often yields substantial symptomatic relief. For example, one long-term series noted that patients’ dyspnoea improved markedly after plication (with nearly half becoming asymptomatic) despite no statistically significant change in FEV1.0 or vital capacity.^22^ Burns and Dunning summarized that a plicated diaphragm leads to better expiratory lung volumes, gas exchange, and exercise capacity in compromised patients, thereby reducing the need for prolonged mechanical ventilation.^23^ Gazala et al.^10^ reported significant improvements in dyspnoea scores and pulmonary function tests when plication was performed several months after the initial phrenic nerve-related surgery, demonstrating the effectiveness of delayed intervention. This disconnect suggests that the benefit of plication is due to mechanical stabilization rather than improved lung volume. By flattening the paralysed hemidiaphragm, plication prevents paradoxical movements and restores more normal thoraco-abdominal mechanics, which alleviates dyspnoea and improves ventilatory efficiency.^16^ Thus, even after right phrenic nerve resection—when objective pulmonary function gains are limited—the stabilizing effect of plication on the chest wall and mediastinum plays a key role in clinical improvement. Further studies with larger cohorts are needed to evaluate both clinical symptoms and outcomes in greater detail.

Post-thoracotomy atrial fibrillation is generally attributed to postoperative inflammation^24^ and ectopic firing from atrial myocardium on pulmonary-vein stumps^25^—mechanisms present in both the plication and non-plication groups. The excess incidence observed only in the non-plication group is more plausibly explained by an additional factor: the elevated, paralysed hemidiaphragm stretches and compresses the atrium and pulmonary veins after phrenic-nerve resection, whereas diaphragmatic plication restores normal position and removes this mechanical trigger.

Although the cohort size and missing pulmonary function data limit statistical power, subgroup analysis of left lobectomy or more extensive resections showed post-hoc powers of 0.447 for d%FVC and 0.471 for d%FEV1.0, with both measures significantly favouring plication. These results suggest a clinically meaningful effect, though larger prospective studies are warranted. In multivariate models adjusting for surgical approach, side, primary disease, and extent of resection, plication continued to show a trend towards smaller declines in d%FVC and d%FEV1.0, and surgical side showed a borderline association with d%FVC (**Table S1**).

This study has several limitations. First, its retrospective, non-randomized design introduces potential selection bias as the decision to perform intraoperative plication was based on each surgeon’s judgement without standardized criteria. Second, the sample size was small, particularly right-sided examples, leading to insufficient statistical power in some analyses. Third, the timing of postoperative pulmonary function tests, approach, patient comorbidities, tumour stage and unmeasured confounders between groups, which may have associated with bias. Fourth, multiple subgroup and secondary analyses were performed without adjustment for multiplicity, increasing the risk of false positive. Analyses were exploratory in nature. Finally, the cohort comprised patients with both lung cancer and thymic epithelial tumours which may limit generalizability. Prospective multicentre trials with predefined criteria and standardized protocols are warranted.

## CONCLUSIONS

Preventive diaphragmatic plication during the initial operation with phrenic nerve resection may help preserve postoperative pulmonary function in patients undergoing lobectomy or more extensive resections of the left lung. However, given the small number of right-sided cases, the retrospective design, and the absence of adjustment for multiple comparisons, these findings should be interpreted with caution. Further prospective studies are needed to validate their generalizability.

## Supplementary Material

ivaf233_Supplementary_Data

## Data Availability

The data will be shared in reasonable request to the corresponding author.
