# Supplementary material for: Intraoperative Diaphragmatic Plication During Initial Surgery With Phrenic Nerve Resection
Source: Interdiscip Cardiovasc Thorac Surg. 2025 Sep 25;40(10):ivaf233. doi: 10.1093/icvts/ivaf233 (PMC12560820; doi:10.1093/icvts/ivaf233)
Supplement: ivaf233_Supplementary_Data [file ivaf233_supplementary_data.zip › Supplementary_Data_20251011/TABLEE1.docx]

Supplementary Table 1 multivariate analysis

| d%FVC |  |  |  |  |
| --- | --- | --- | --- | --- |
| **Variable** | **Estimate (β)** | **Std. Error** | **t value** | **p value** |
| (Intercept) | –7.36 | 7.21 | –1.02 | 0.312 |
| Extent of resection (≥ lobectomy vs ≤ segmentectomy) | –2.10 | 1.53 | –1.37 | 0.175 |
| Diaphragm plication (yes vs no) | –6.47 | 3.72 | –1.74 | 0.087 |
| Surgical approach (Posterolateral thoracotomy/hemi-clamshell vs other) | –0.91 | 1.61 | –0.57 | 0.574 |
| Primary disease (lung cancer vs thymic tumor) | –0.89 | 5.92 | –0.15 | 0.881 |
| Surgical side (left vs right) | –6.46 | 3.82 | –1.69 | 0.095 |
|  |  |  |  |  |
| d%FEV1.0 |  |  |  |  |
| **Variable** | **Estimate (β)** | **Std. Error** | **t value** | **p value** |
| (Intercept) | –15.52 | 7.26 | –2.14 | 0.036* |
| Extent of resection (≥ lobectomy vs ≤ segmentectomy) | 0.04 | 1.54 | 0.03 | 0.978 |
| Diaphragm plication (yes vs no) | –7.11 | 3.75 | –1.90 | 0.062· |
| Surgical approach (Posterolateral thoracotomy/hemi-clamshell vs other) | –0.96 | 1.62 | –0.60 | 0.554 |
| Primary disease (lung cancer vs thymic tumor) | –5.28 | 5.96 | –0.89 | 0.379 |
| Surgical side (left vs right) | –3.25 | 3.84 | –0.85 | 0.401 |
