# Supplementary figures and images for: Intraoperative Diaphragmatic Plication During Initial Surgery With Phrenic Nerve Resection
Source: Interdiscip Cardiovasc Thorac Surg. 2025 Sep 25;40(10):ivaf233. doi: 10.1093/icvts/ivaf233 (PMC12560820; doi:10.1093/icvts/ivaf233)

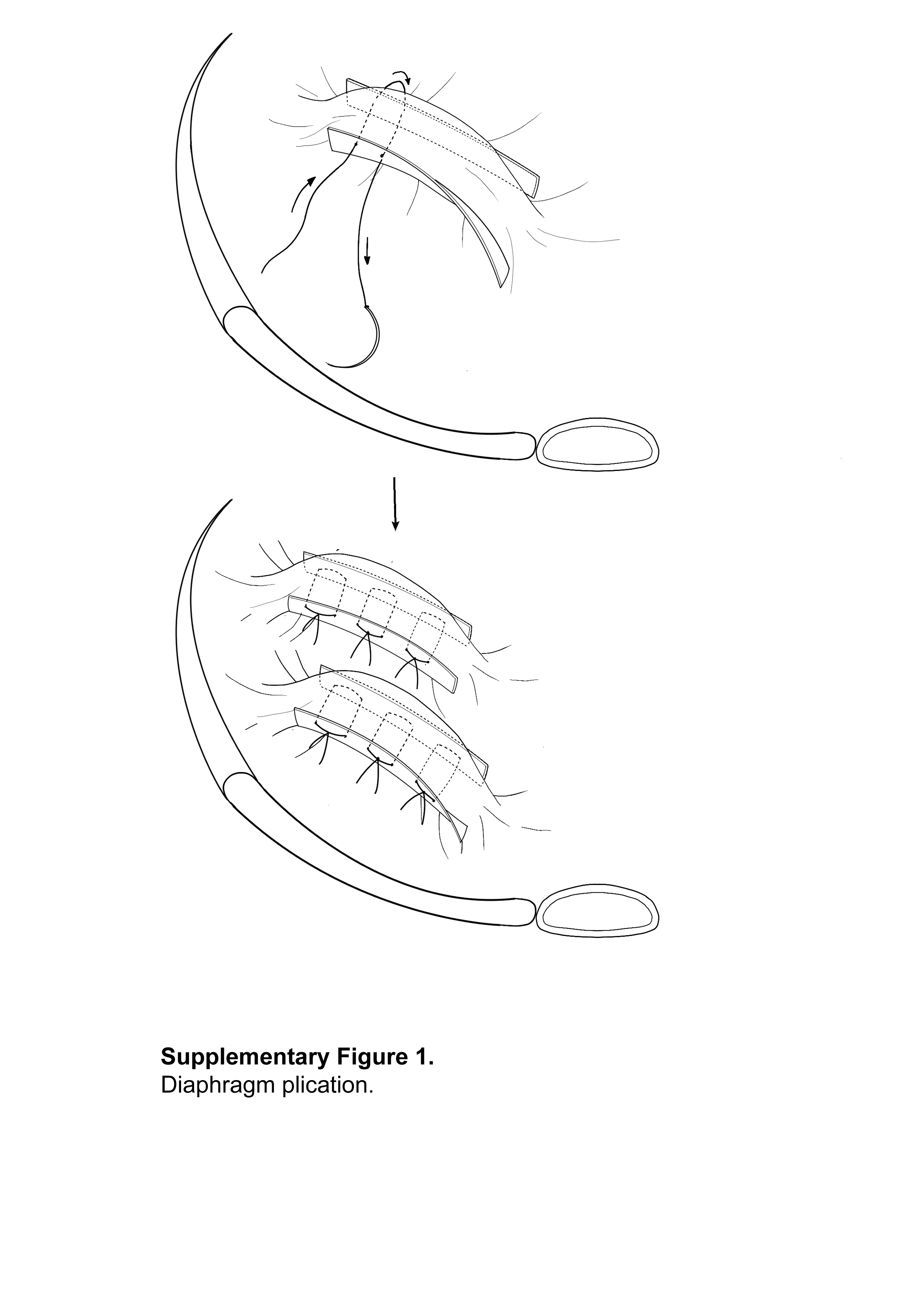

Supplement: ivaf233_Supplementary_Data [file ivaf233_supplementary_data.zip › Supplementary_Data_20251011/FIGE1.tif]

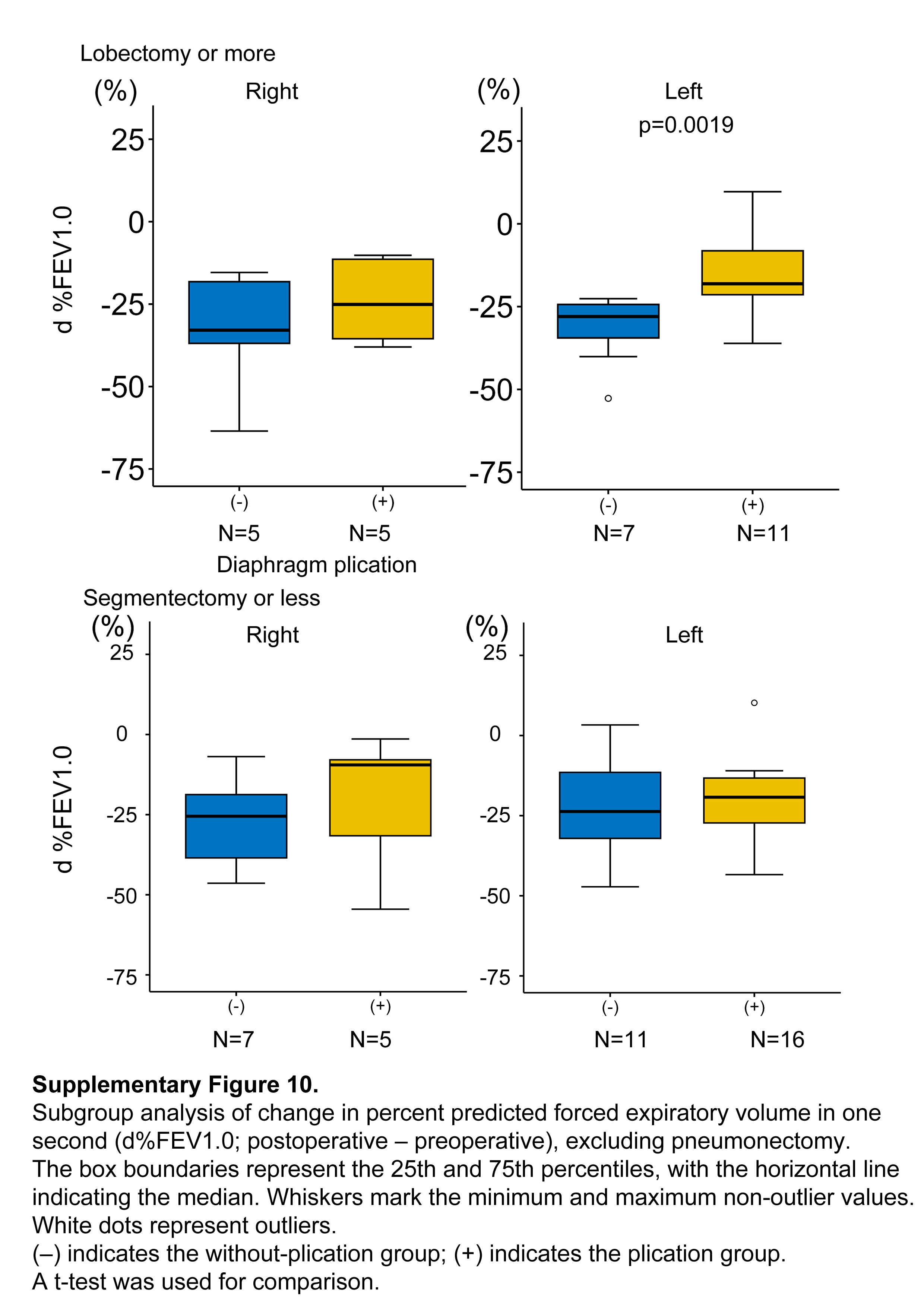

Supplement: ivaf233_Supplementary_Data [file ivaf233_supplementary_data.zip › Supplementary_Data_20251011/FIGE10.tif]

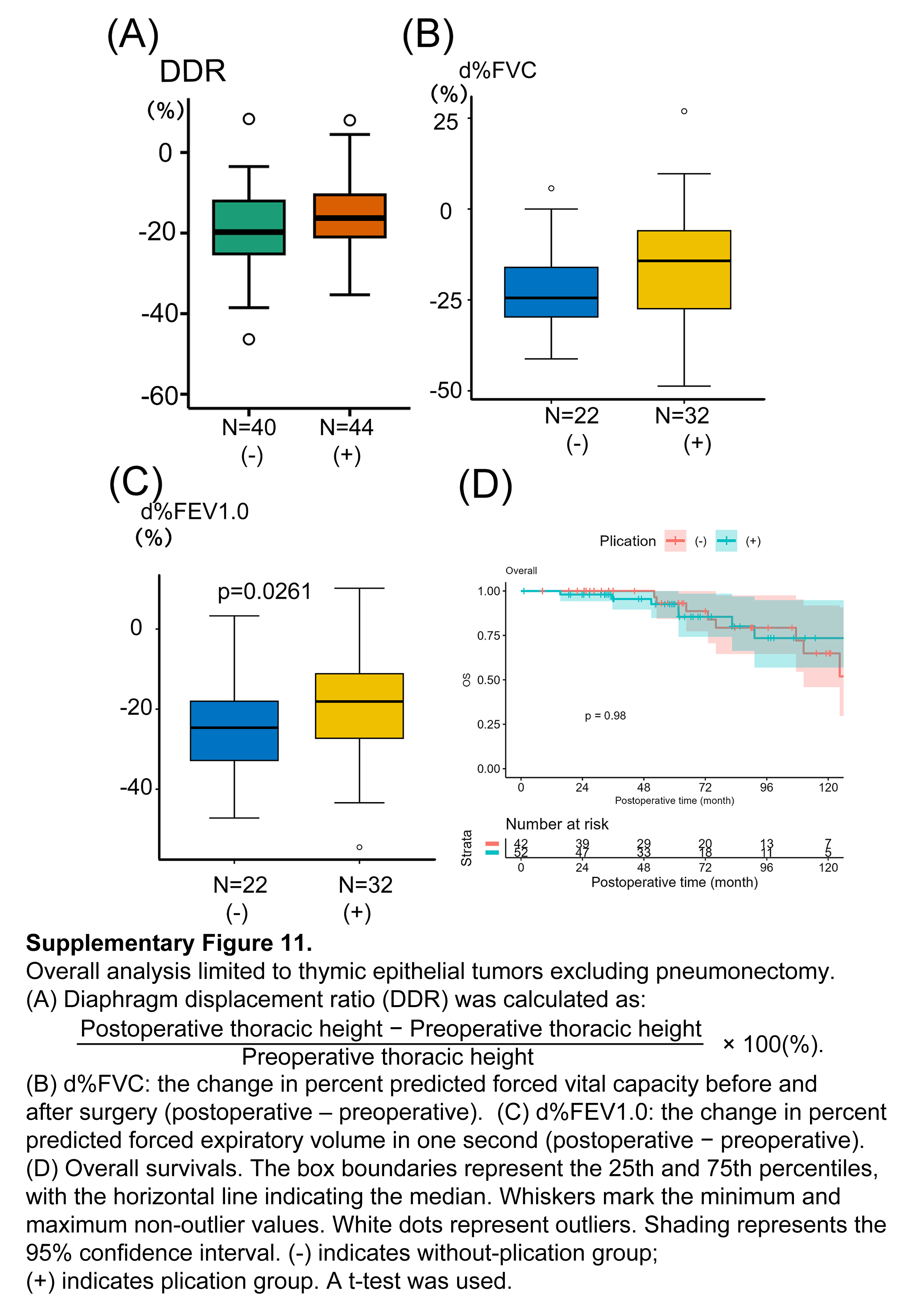

Supplement: ivaf233_Supplementary_Data [file ivaf233_supplementary_data.zip › Supplementary_Data_20251011/FIGE11.tif]

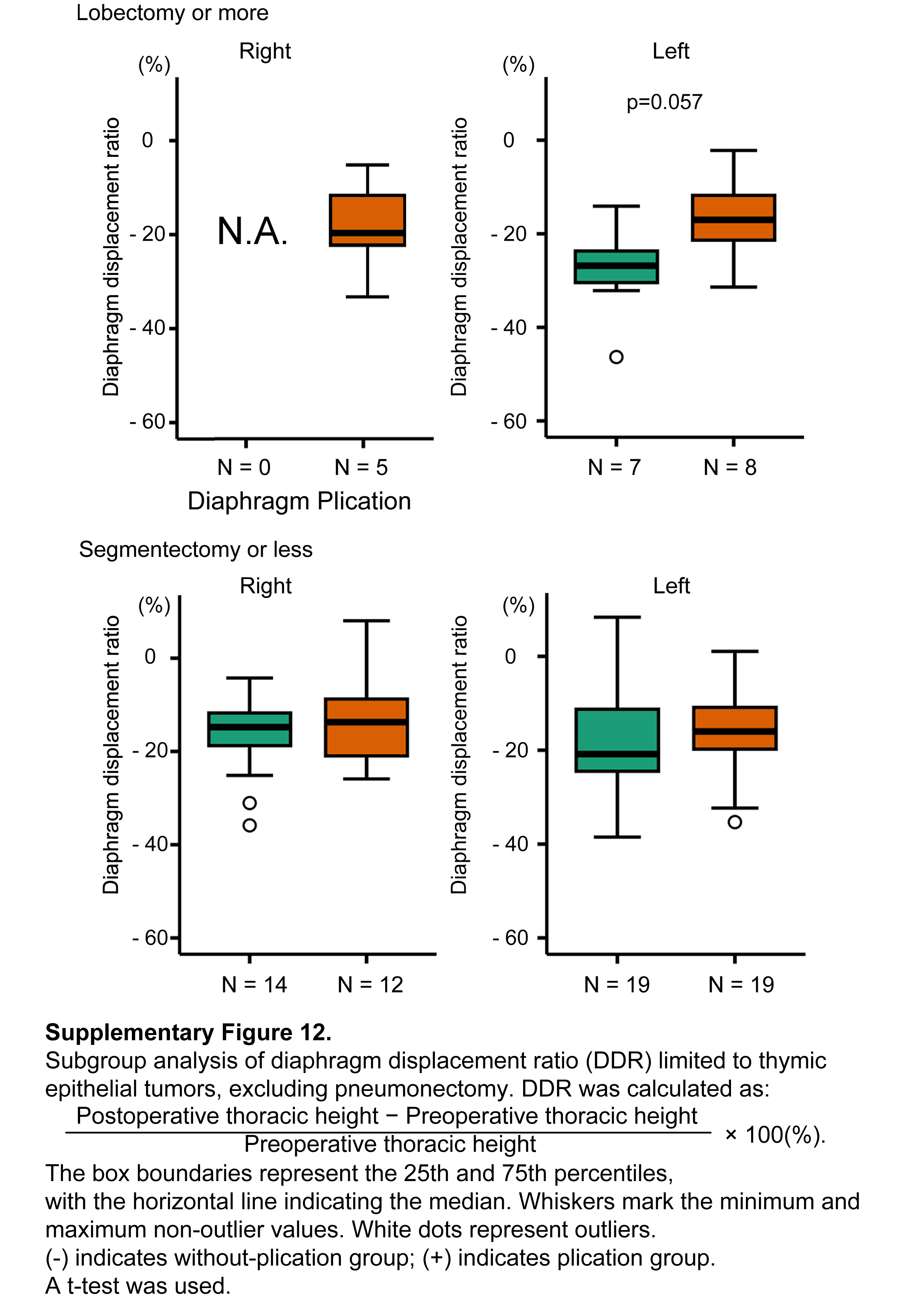

Supplement: ivaf233_Supplementary_Data [file ivaf233_supplementary_data.zip › Supplementary_Data_20251011/FIGE12.tif]

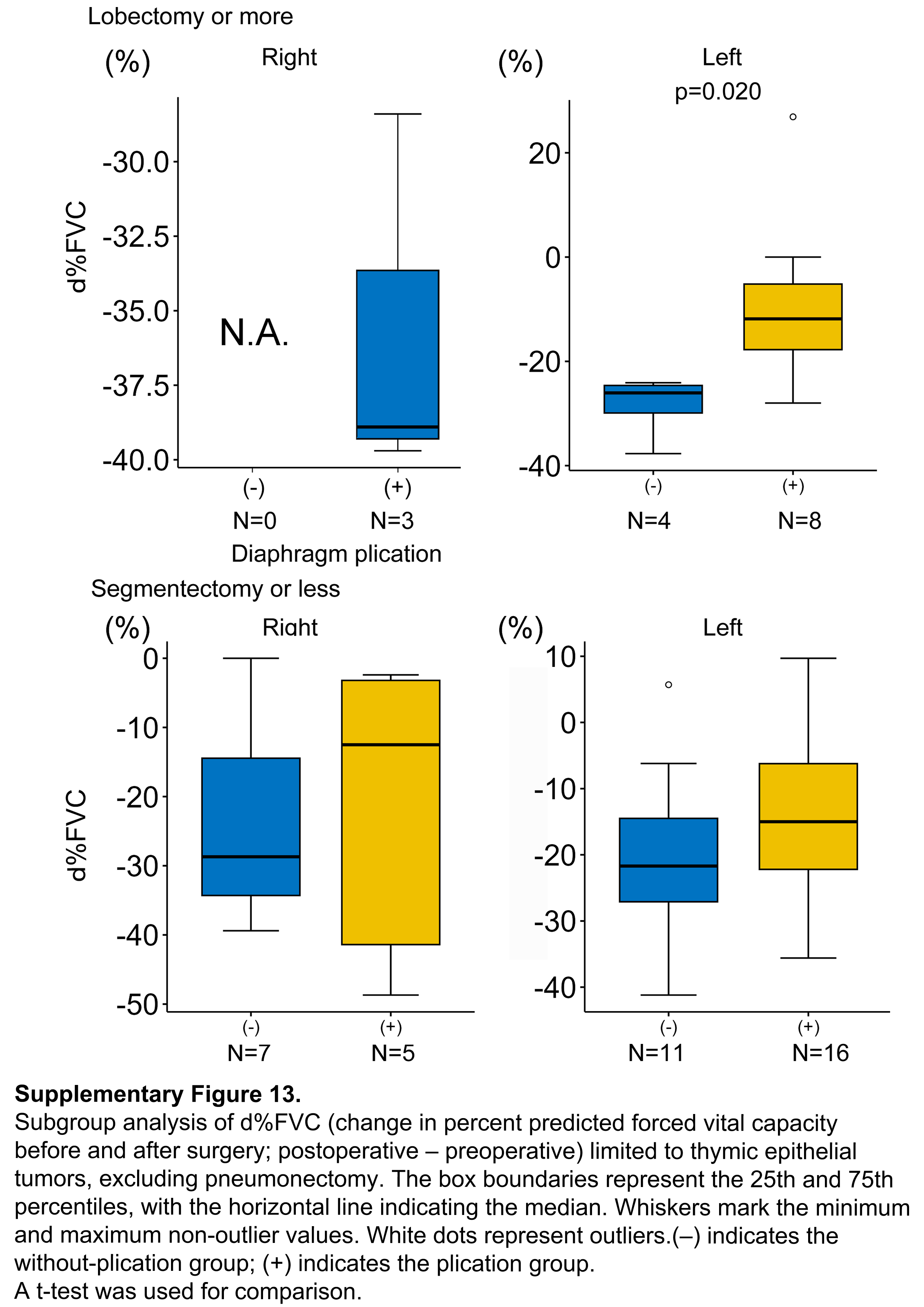

Supplement: ivaf233_Supplementary_Data [file ivaf233_supplementary_data.zip › Supplementary_Data_20251011/FIGE13.tif]

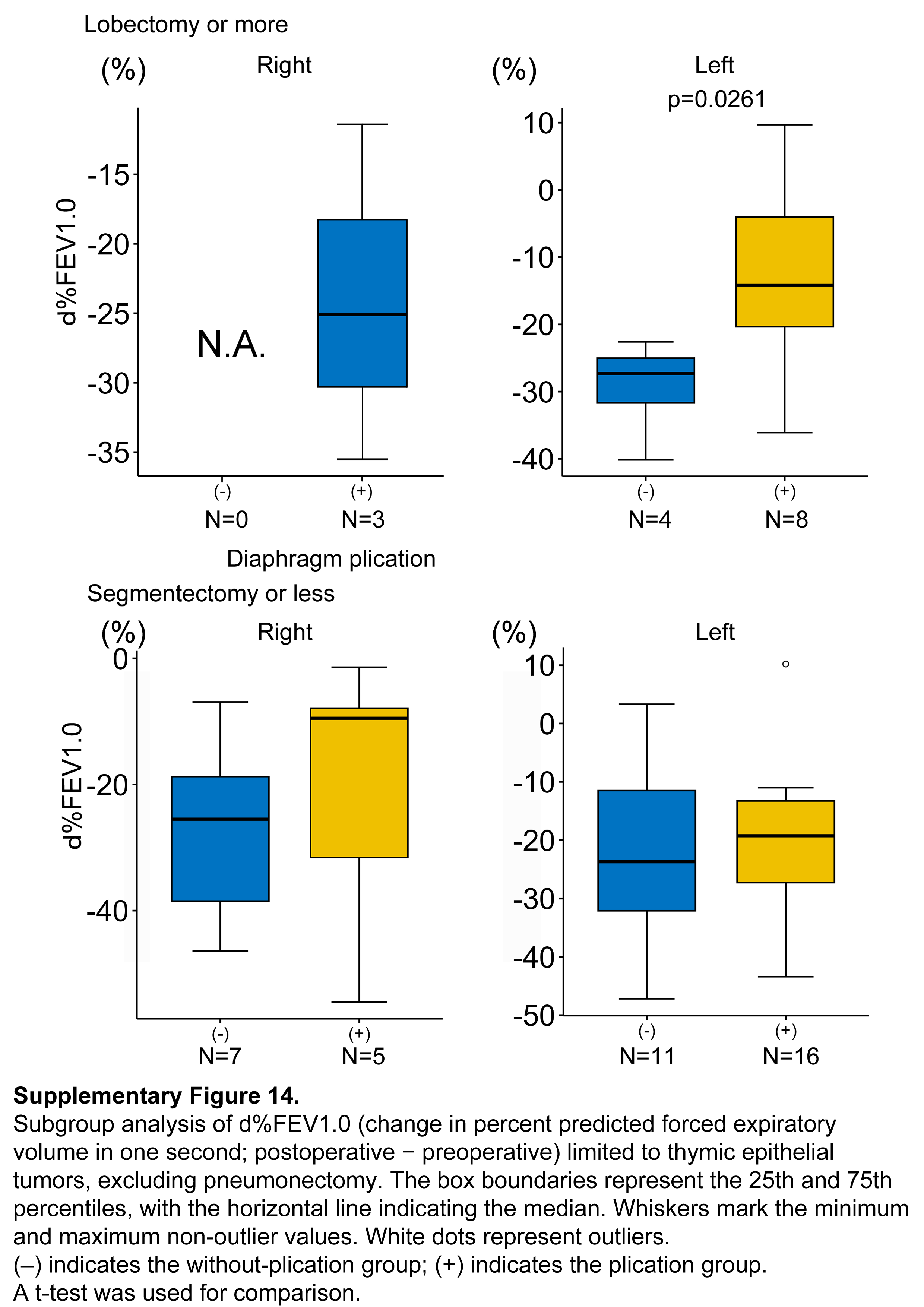

Supplement: ivaf233_Supplementary_Data [file ivaf233_supplementary_data.zip › Supplementary_Data_20251011/FIGE14.tif]

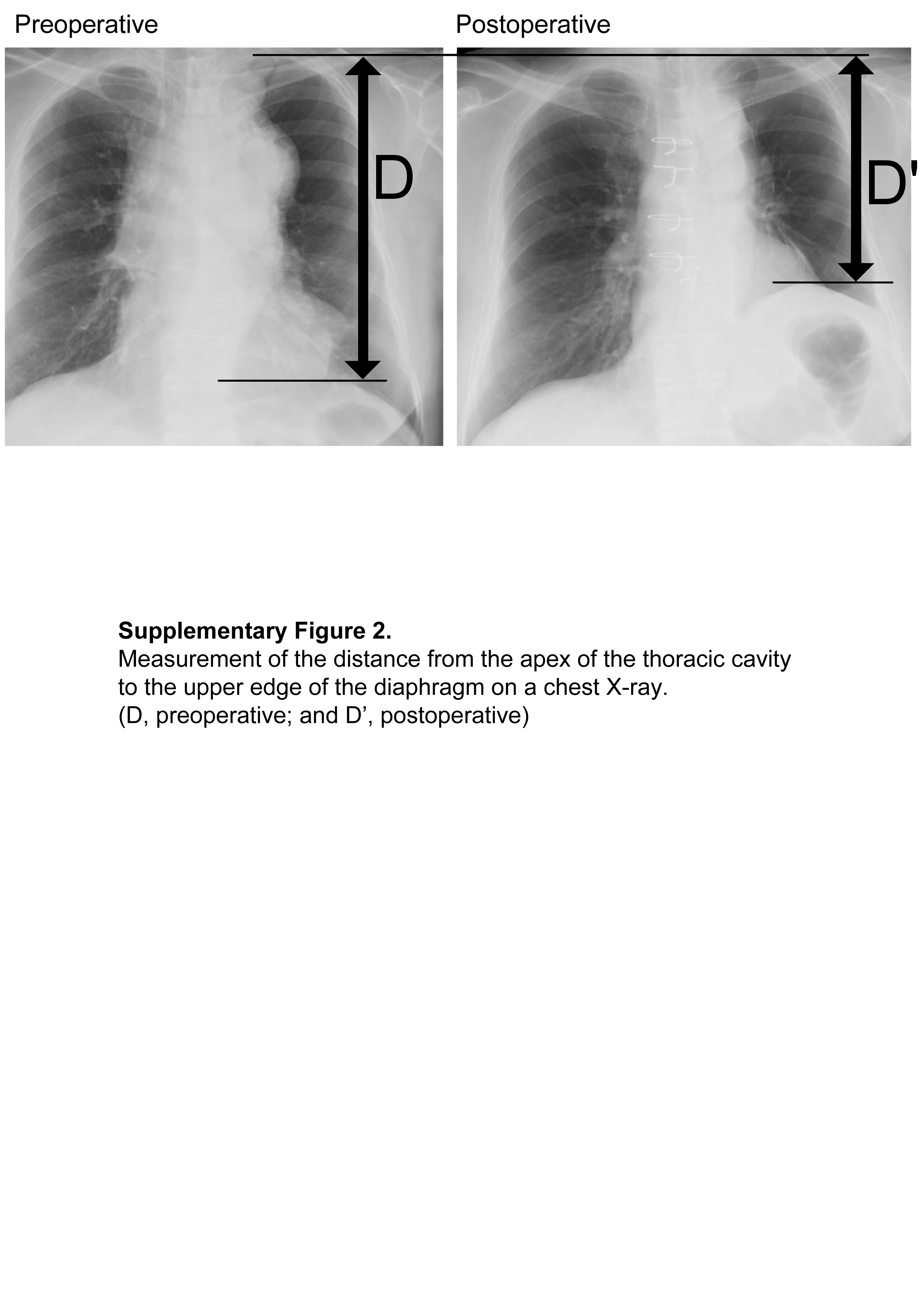

Supplement: ivaf233_Supplementary_Data [file ivaf233_supplementary_data.zip › Supplementary_Data_20251011/FIGE2.tif]

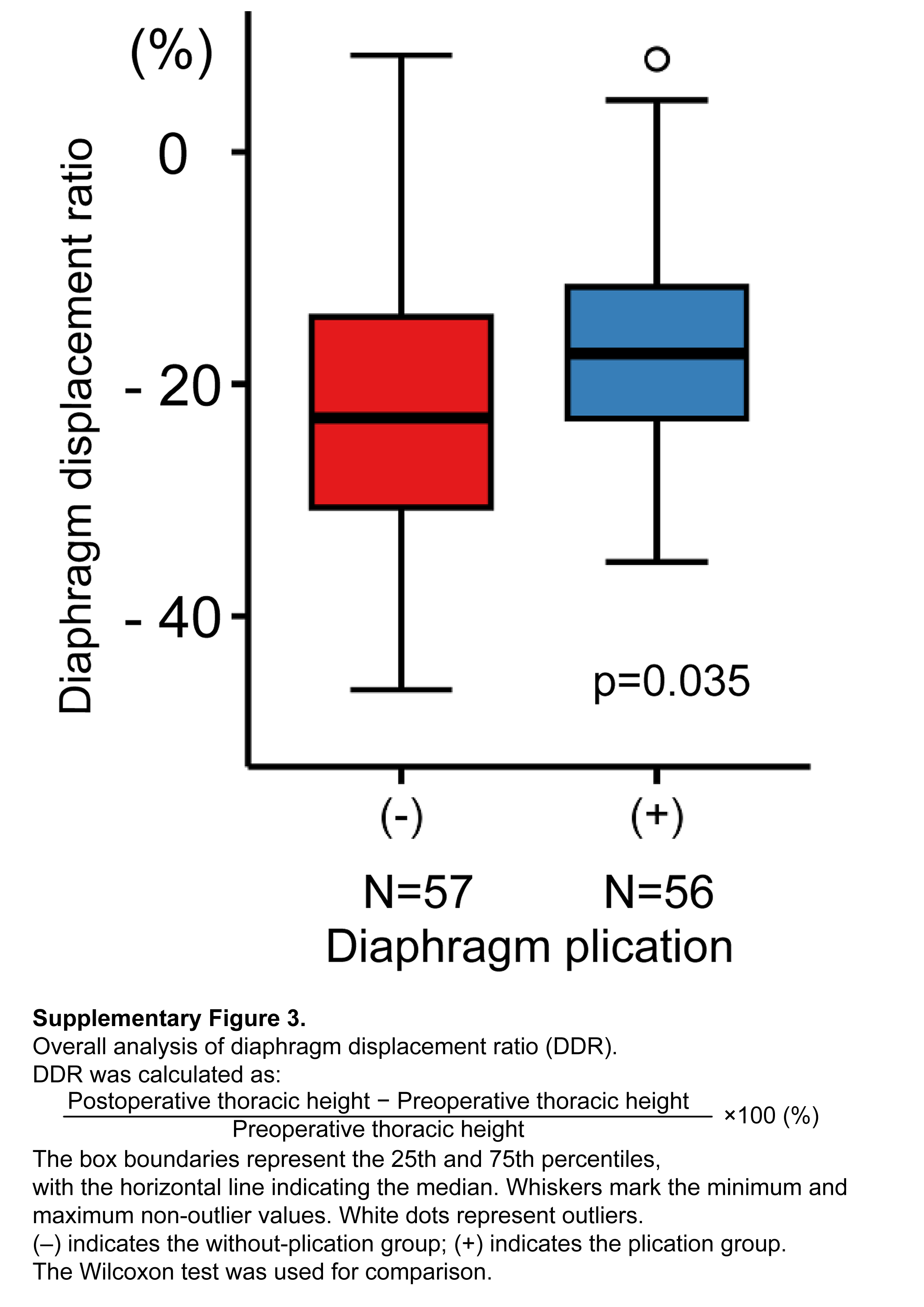

Supplement: ivaf233_Supplementary_Data [file ivaf233_supplementary_data.zip › Supplementary_Data_20251011/FIGE3.tif]

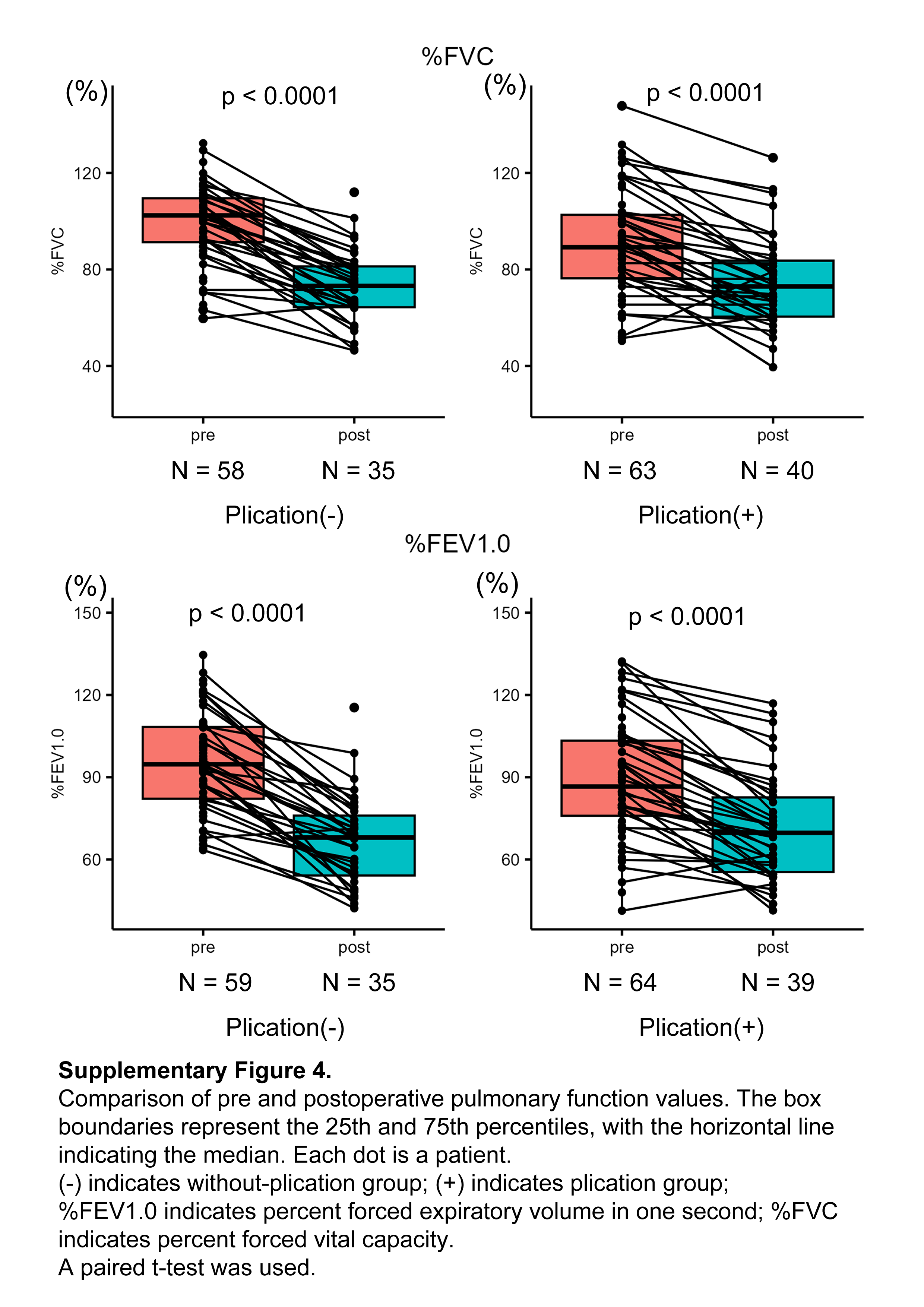

Supplement: ivaf233_Supplementary_Data [file ivaf233_supplementary_data.zip › Supplementary_Data_20251011/FIGE4.tif]

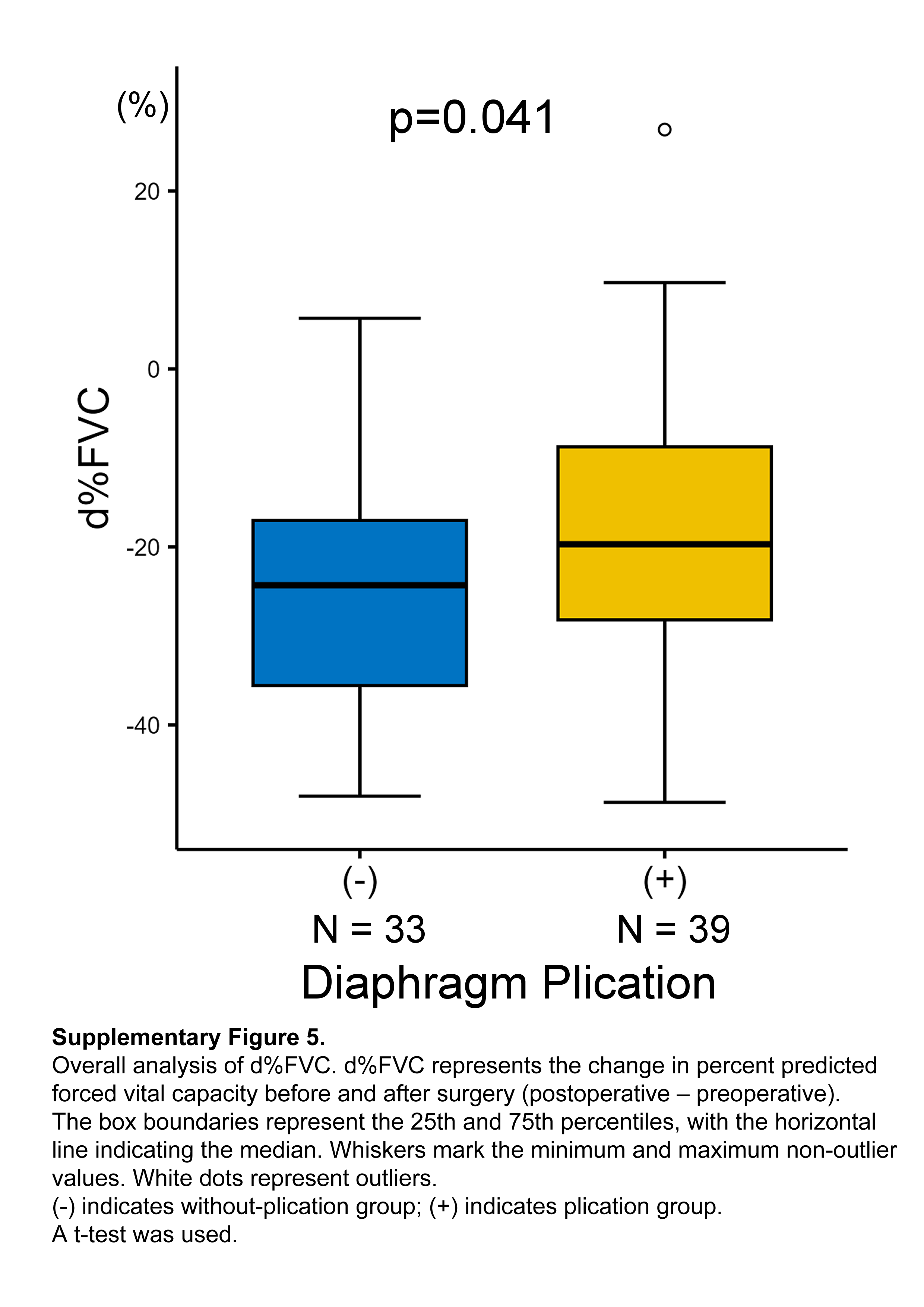

Supplement: ivaf233_Supplementary_Data [file ivaf233_supplementary_data.zip › Supplementary_Data_20251011/FIGE5.tif]

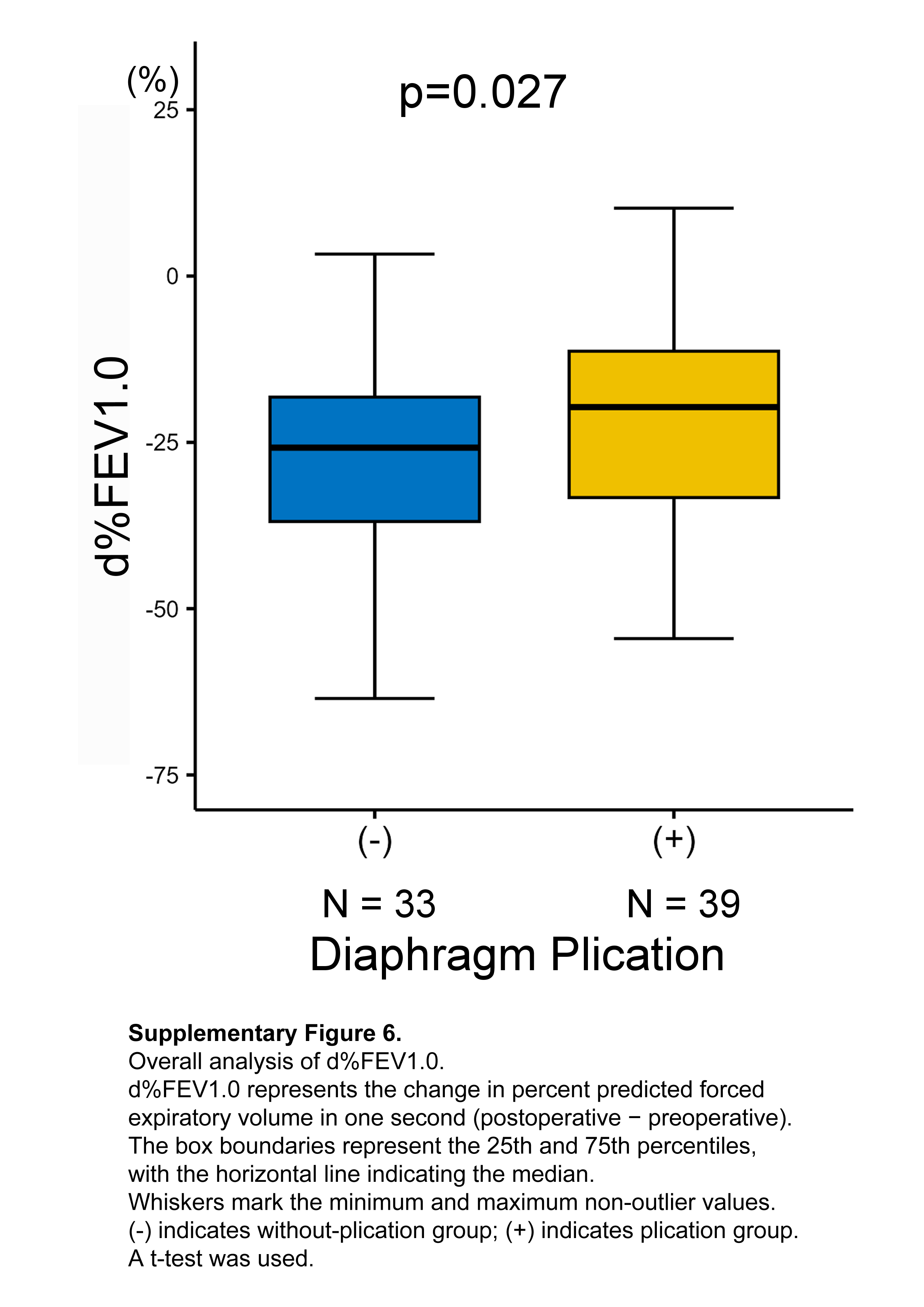

Supplement: ivaf233_Supplementary_Data [file ivaf233_supplementary_data.zip › Supplementary_Data_20251011/FIGE6.tif]

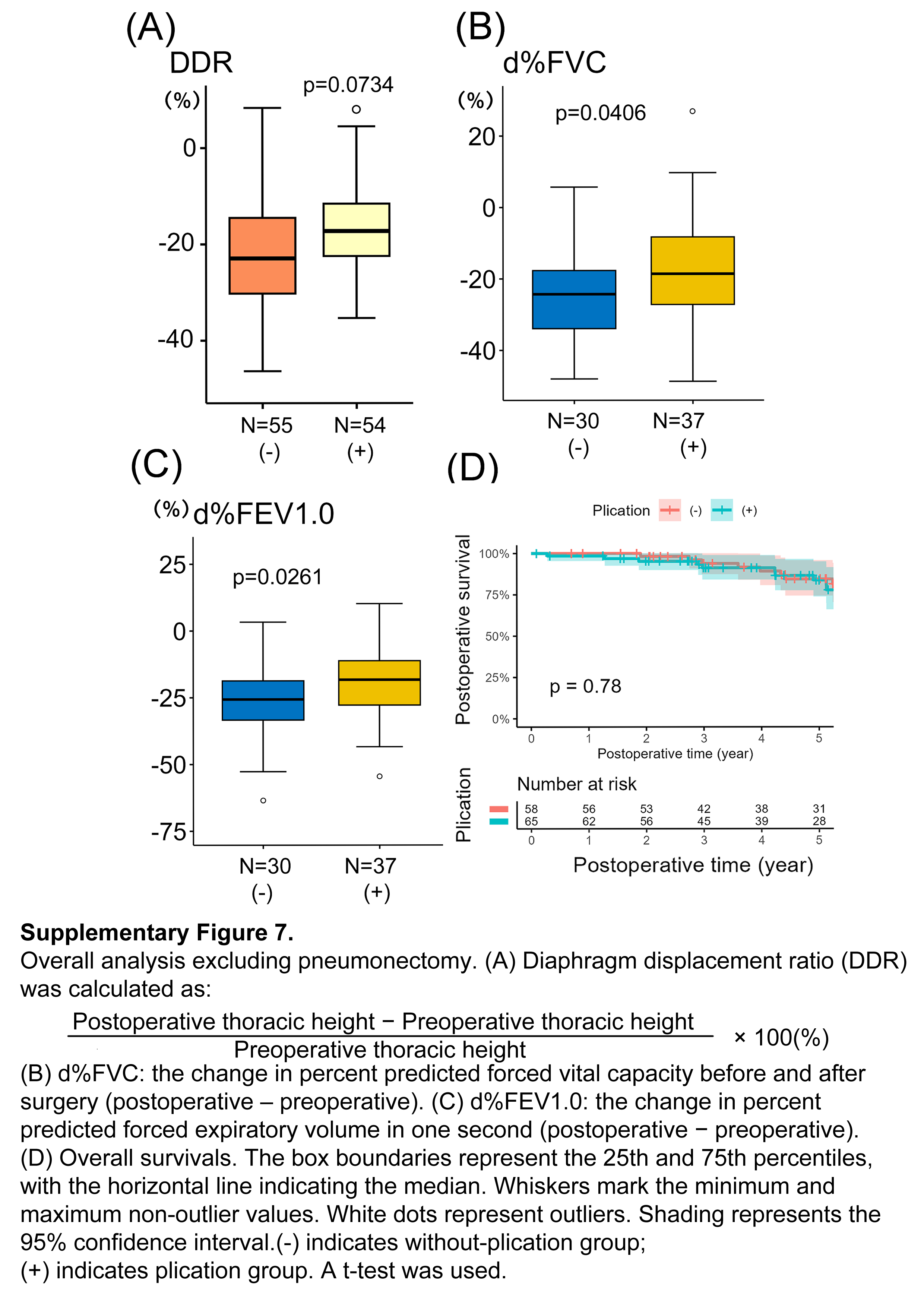

Supplement: ivaf233_Supplementary_Data [file ivaf233_supplementary_data.zip › Supplementary_Data_20251011/FIGE7.tif]

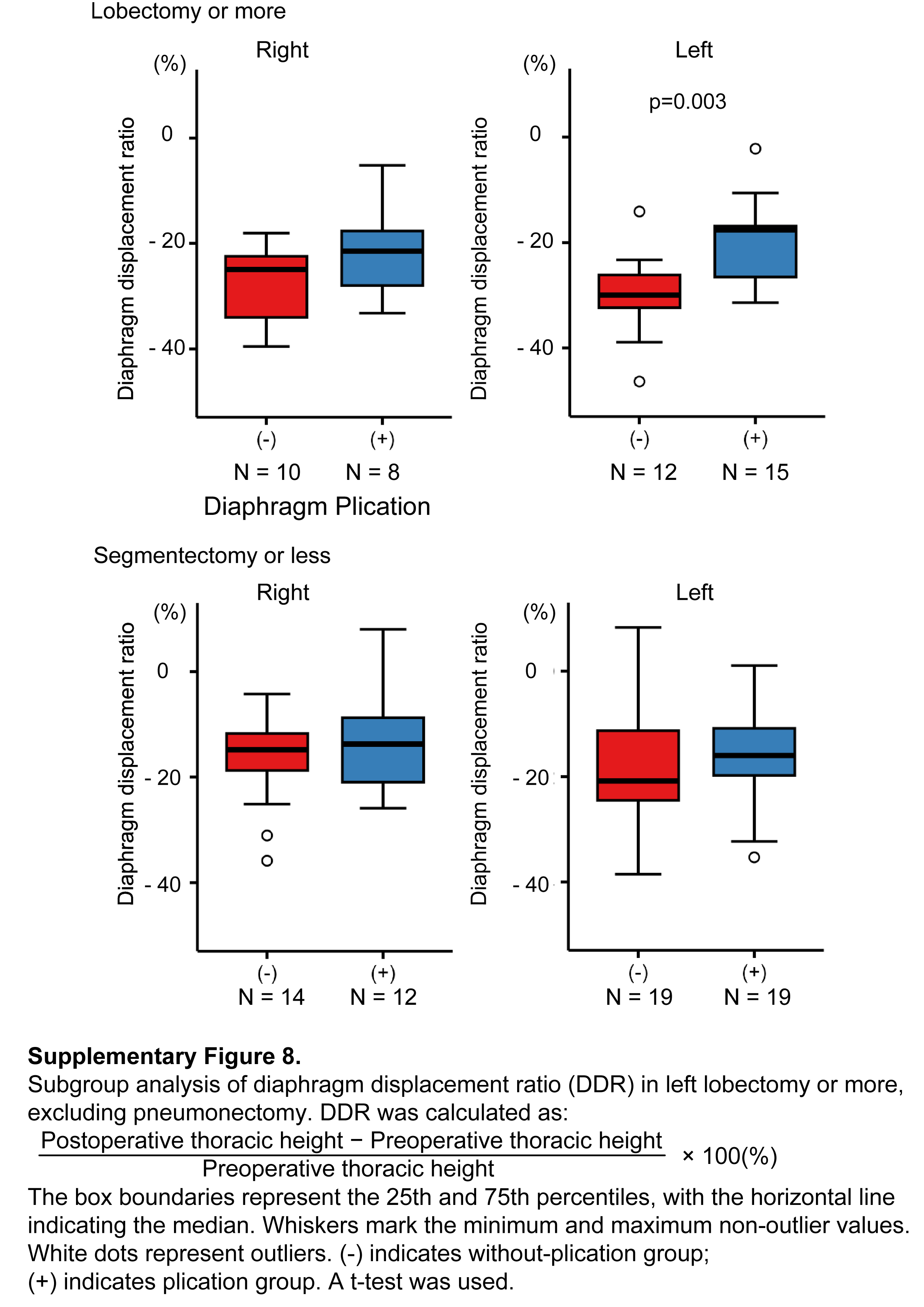

Supplement: ivaf233_Supplementary_Data [file ivaf233_supplementary_data.zip › Supplementary_Data_20251011/FIGE8.tif]

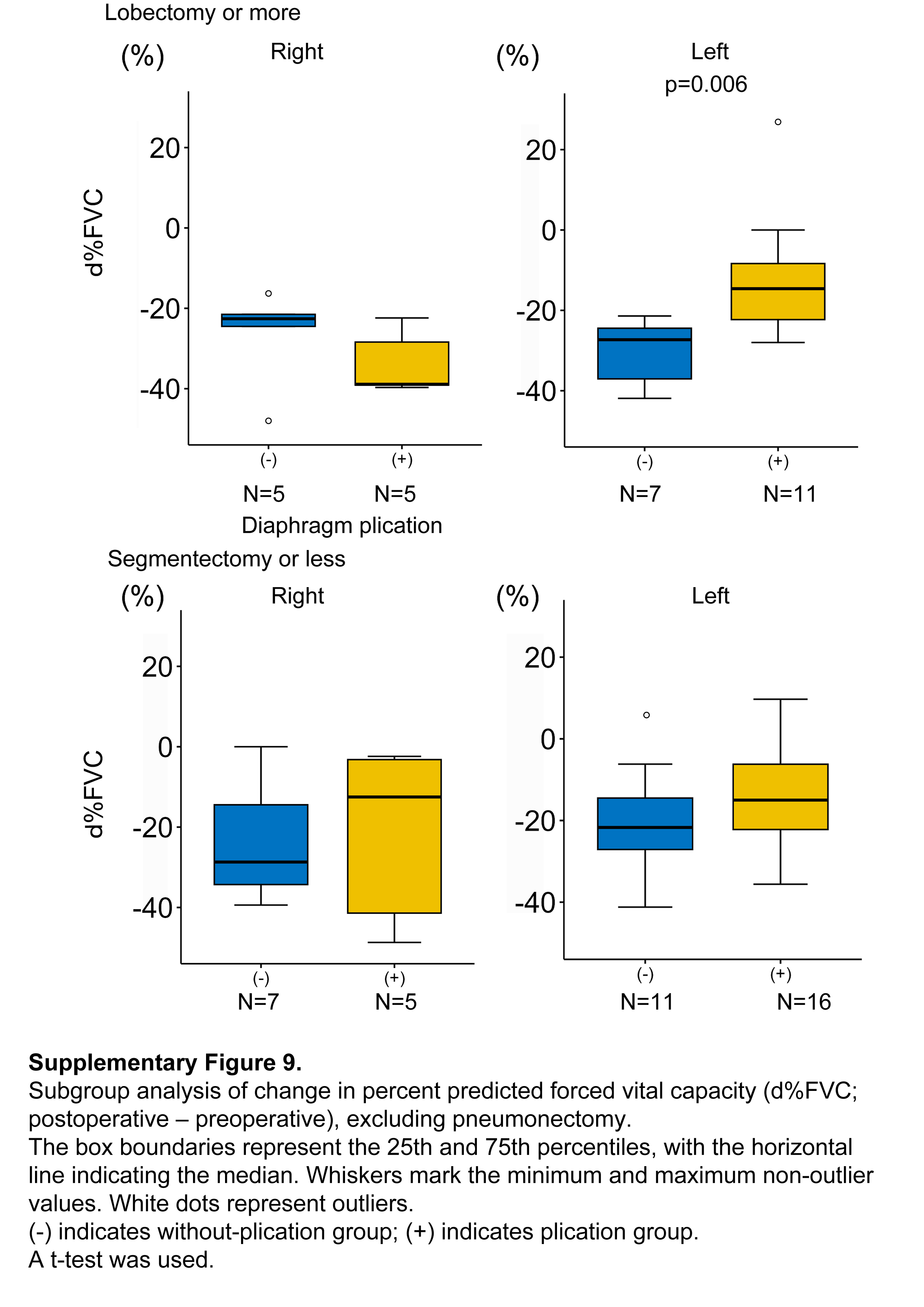

Supplement: ivaf233_Supplementary_Data [file ivaf233_supplementary_data.zip › Supplementary_Data_20251011/FIGE9.tif]
